# Supplementary material for: HIVprotI: an integrated web based platform for prediction and design of HIV proteins inhibitors
Source: J Cheminform. 2018 Mar 9;10:12. doi: 10.1186/s13321-018-0266-y (PMC5845081; doi:10.1186/s13321-018-0266-y)
Supplement: Supplementary file 2 — Additional file 2. Source code of HIVProtI web server. [file 13321_2018_266_MOESM2_ESM.zip › HIVprotI_Source-code/hivprotI/weka-3-6-6/doc/weka/filters/unsupervised/attribute/PartitionedMultiFilter.html]

PartitionedMultiFilter


---


|  |  |  |
| --- | --- | --- |
| **Overview** \| **Package** \| **Class** \| **Tree** \| **Deprecated** \| **Index** \| **Help** \| **Weka's home** | |  |
| **PREV CLASS**   **NEXT CLASS** | **FRAMES**    **NO FRAMES**     **All Classes** |  |
| SUMMARY: NESTED \| FIELD \| CONSTR \| METHOD | DETAIL: FIELD \| CONSTR \| METHOD |  |


---


## weka.filters.unsupervised.attribute Class PartitionedMultiFilter

```
java.lang.Object
  weka.filters.Filter
      weka.filters.SimpleFilter
          weka.filters.SimpleBatchFilter
              weka.filters.unsupervised.attribute.PartitionedMultiFilter
```

**All Implemented Interfaces:**: java.io.Serializable, CapabilitiesHandler, OptionHandler, RevisionHandler

---

``` public class PartitionedMultiFilter extends SimpleBatchFilter ```

A filter that applies filters on subsets of attributes and assembles the output into a new dataset. Attributes that are not covered by any of the ranges can be either retained or removed from the output.


Valid options are:

```
 -D
  Turns on output of debugging information.
```

```
 -F <classname [options]>
  A filter to apply (can be specified multiple times).
```

```
 -R <range>
  An attribute range (can be specified multiple times).
  For each filter a range must be supplied. 'first' and 'last'
  are valid indices. 'inv(...)' around the range denotes an
  inverted range.
```

```
 -U
  Flag for leaving unused attributes out of the output, by default
  these are included in the filter output.
```

**Version:**
:   $Revision: 7506 $

**Author:**
:   FracPete (fracpete at waikato dot ac dot nz)

**See Also:**: `StreamableFilter`, Serialized Form

---

**Constructor Summary**

`PartitionedMultiFilter()`


| **Method Summary** | |
| --- | --- |
| `java.lang.String` | `filtersTipText()`             Returns the tip text for this property. |
| `Filter` | `getFilter(int index)`             Gets a single filter from the set of available filters. |
| `Filter[]` | `getFilters()`             Gets the list of possible filters to choose from. |
| `java.lang.String[]` | `getOptions()`             Gets the current settings of the filter. |
| `Range` | `getRange(int index)`             Gets a single Range from the set of available Ranges. |
| `Range[]` | `getRanges()`             Gets the list of possible Ranges to choose from. |
| `boolean` | `getRemoveUnused()`             Gets whether unused attributes (ones that are not covered by any of the ranges) are removed from the output. |
| `java.lang.String` | `getRevision()`             Returns the revision string. |
| `java.lang.String` | `globalInfo()`             Returns a string describing this filter. |
| `java.util.Enumeration` | `listOptions()`             Returns an enumeration describing the available options. |
| `static void` | `main(java.lang.String[] args)`             Main method for executing this class. |
| `java.lang.String` | `rangesTipText()`             Returns the tip text for this property. |
| `java.lang.String` | `removeUnusedTipText()`             Returns the tip text for this property. |
| `void` | `setFilters(Filter[] filters)`             Sets the list of possible filters to choose from. |
| `void` | `setOptions(java.lang.String[] options)`             Parses a list of options for this object. |
| `void` | `setRanges(Range[] Ranges)`             Sets the list of possible Ranges to choose from. |
| `void` | `setRemoveUnused(boolean value)`             Sets whether unused attributes (ones that are not covered by any of the ranges) are removed from the output. |

**Methods inherited from class weka.filters.SimpleBatchFilter**

`batchFinished, input`

**Methods inherited from class weka.filters.SimpleFilter**

`debugTipText, getDebug, setDebug, setInputFormat`

**Methods inherited from class weka.filters.Filter**

`batchFilterFile, filterFile, getCapabilities, getCapabilities, getOutputFormat, isFirstBatchDone, isNewBatch, isOutputFormatDefined, makeCopies, makeCopy, numPendingOutput, output, outputPeek, toString, useFilter, wekaStaticWrapper`

**Methods inherited from class java.lang.Object**

`equals, getClass, hashCode, notify, notifyAll, wait, wait, wait`

**Constructor Detail**

### PartitionedMultiFilter

```
public PartitionedMultiFilter()
```


**Method Detail**

### globalInfo

```
public java.lang.String globalInfo()
```

:   Returns a string describing this filter.

    :   **Specified by:**: `globalInfo` in class `SimpleFilter`
    :   **Returns:**: a description of the filter suitable for displaying in the explorer/experimenter gui

---


### listOptions

```
public java.util.Enumeration listOptions()
```

:   Returns an enumeration describing the available options.

    :   **Specified by:**: `listOptions` in interface `OptionHandler` **Overrides:**: `listOptions` in class `SimpleFilter`
    :   **Returns:**: an enumeration of all the available options.

---


### setOptions

```
public void setOptions(java.lang.String[] options)
                throws java.lang.Exception
```

:   Parses a list of options for this object.


    Valid options are:

    ```
     -D
      Turns on output of debugging information.
    ```

    ```
     -F <classname [options]>
      A filter to apply (can be specified multiple times).
    ```

    ```
     -R <range>
      An attribute range (can be specified multiple times).
      For each filter a range must be supplied. 'first' and 'last'
      are valid indices. 'inv(...)' around the range denotes an
      inverted range.
    ```

    ```
     -U
      Flag for leaving unused attributes out of the output, by default
      these are included in the filter output.
    ```

    :   **Specified by:**: `setOptions` in interface `OptionHandler` **Overrides:**: `setOptions` in class `SimpleFilter`
    :   **Parameters:**: `options` - the list of options as an array of strings **Throws:**: `java.lang.Exception` - if an option is not supported **See Also:**: `SimpleFilter.reset()`

---


### getOptions

```
public java.lang.String[] getOptions()
```

:   Gets the current settings of the filter.

    :   **Specified by:**: `getOptions` in interface `OptionHandler` **Overrides:**: `getOptions` in class `SimpleFilter`
    :   **Returns:**: an array of strings suitable for passing to setOptions

---


### setRemoveUnused

```
public void setRemoveUnused(boolean value)
```

:   Sets whether unused attributes (ones that are not covered by any of the
    ranges) are removed from the output.

    :   **Parameters:**: `value` - if true then the unused attributes get removed

---


### getRemoveUnused

```
public boolean getRemoveUnused()
```

:   Gets whether unused attributes (ones that are not covered by any of the
    ranges) are removed from the output.

    :   **Returns:**: true if unused attributes are removed

---


### removeUnusedTipText

```
public java.lang.String removeUnusedTipText()
```

:   Returns the tip text for this property.

    :   **Returns:**: tip text for this property suitable for displaying in the explorer/experimenter gui

---


### setFilters

```
public void setFilters(Filter[] filters)
```

:   Sets the list of possible filters to choose from.
    Also resets the state of the filter (this reset doesn't affect the
    options).

    :   **Parameters:**: `filters` - an array of filters with all options set. **See Also:**: `SimpleFilter.reset()`

---


### getFilters

```
public Filter[] getFilters()
```

:   Gets the list of possible filters to choose from.

    :   **Returns:**: the array of Filters

---


### filtersTipText

```
public java.lang.String filtersTipText()
```

:   Returns the tip text for this property.

    :   **Returns:**: tip text for this property suitable for displaying in the explorer/experimenter gui

---


### getFilter

```
public Filter getFilter(int index)
```

:   Gets a single filter from the set of available filters.

    :   **Parameters:**: `index` - the index of the filter wanted **Returns:**: the Filter

---


### setRanges

```
public void setRanges(Range[] Ranges)
```

:   Sets the list of possible Ranges to choose from.
    Also resets the state of the Range (this reset doesn't affect the
    options).

    :   **Parameters:**: `Ranges` - an array of Ranges with all options set. **See Also:**: `SimpleFilter.reset()`

---


### getRanges

```
public Range[] getRanges()
```

:   Gets the list of possible Ranges to choose from.

    :   **Returns:**: the array of Ranges

---


### rangesTipText

```
public java.lang.String rangesTipText()
```

:   Returns the tip text for this property.

    :   **Returns:**: tip text for this property suitable for displaying in the explorer/experimenter gui

---


### getRange

```
public Range getRange(int index)
```

:   Gets a single Range from the set of available Ranges.

    :   **Parameters:**: `index` - the index of the Range wanted **Returns:**: the Range

---


### getRevision

```
public java.lang.String getRevision()
```

:   Returns the revision string.

    :   **Specified by:**: `getRevision` in interface `RevisionHandler` **Overrides:**: `getRevision` in class `Filter`
    :   **Returns:**: the revision

---


### main

```
public static void main(java.lang.String[] args)
```

:   Main method for executing this class.

    :   **Parameters:**: `args` - should contain arguments for the filter: use -h for help


---


|  |  |  |
| --- | --- | --- |
| **Overview** \| **Package** \| **Class** \| **Tree** \| **Deprecated** \| **Index** \| **Help** \| **Weka's home** | |  |
| **PREV CLASS**   **NEXT CLASS** | **FRAMES**    **NO FRAMES**     **All Classes** |  |
| SUMMARY: NESTED \| FIELD \| CONSTR \| METHOD | DETAIL: FIELD \| CONSTR \| METHOD |  |


---
